# The association between coffee intake and femoral neck bone mineral density based on the NHANES and Mendelian randomisation study

**DOI:** 10.1017/jns.2024.38

**Published:** 2025-07-18

**Authors:** Ke Wang, Guoxin Huang, Ying Liu, Beibei Zhang, Da Qian, Bin Pei

**Affiliations:** 1 Department of Burn and Plastic Surgery-Hand Surgery, Changshu Hospital Affiliated to Soochow University, Changshu No.1 People’s Hospital, Changshu, China; 2 Department of Evidence-Based Medicine Center, Xiangyang No.1 People’s Hospital, Hubei University of Medicine, Xiangyang, China; 3 School of Public Health and Management, Hubei University of Medicine, Shiyan, China; 4 Chronic Disease Department of Xiangyang Center for Disease Control and Prevention, Xiangyang, China

**Keywords:** Coffee, Femoral neck bone mineral density, Mendelian randomisation, National Health and Nutrition Examination Survey

## Abstract

Femoral neck bone mineral density (FNBMD) is a high risk factor for femoral head fractures, and coffee intake affects bone mineral density, but the effect on FNBMD remains to be explored. First, we conducted an observational study in the National Health and Nutrition Examination Survey and collected data on coffee intake, FNBMD, and sixteen covariates. Weight linear regression was used to explore the association of coffee intake with FNBMD. Then, Mendelian randomisation (MR) was used to explore the causal relationship between coffee intake and FNBMD, the exposure factor was coffee intake, and the outcome factor was FNBMD. The inverse variance weighting (IVW) method was used for the analysis, while heterogeneity tests, sensitivity, and pleiotropy analysis were performed. A total of 5 915 people were included in the cross-sectional study, including 3 178 men and 2 737 women. In the completely adjusted model, no coffee was used as a reference. The ORs for the overall population at ‘< 1’, ‘1–<2’, ‘2–<4’, and ‘4+’ (95% CI) were 0.02 (–0.01, 0.04), 0.00 (–0.01, 0.02), –0.01 (–0.02, 0.00), and 0.00 (–0.01, 0.02), respectively. The male and female population showed no statistically significant differences in both univariate and multivariate linear regressions. In the MR study, the IVW results showed an OR (95% CI) of 1.06 (0.88–1.27), a *P*-value of 0.55, and an overall *F*-value of 80.31. The heterogeneity, sensitivity analyses, and pleiotropy had no statistical significance. Our study used cross-sectional studies and MR to demonstrate that there is no correlation or causal relationship between coffee intake and FNBMD.

## Introduction

Coffee is one of the world’s most popular beverages and one of the main sources of caffeine.^(1)^ Coffee has antioxidant and anti-inflammatory properties, regulates dopaminergic transmission, promotes the release of serotonin, and contains a variety of bioactive compounds, such as chlorogenic acid, catechins, magnesium, lignan, and fenugreek, among other substances.^(2)^ Previous studies have found a correlation between coffee and a variety of diseases; for example, coffee intake is protective against CVD and depression^(3,4)^ but is a risk factor for diseases such as respiratory disease and diabetes,^(5,6)^ while its effect on bone mineral density (BMD) is not yet clear. BMD is an important indicator of bone strength that is usually measured by dual-energy X-ray absorptiometry, which directly reflects the degree of osteoporosis.^(7)^ Femoral neck bone mineral density (FNBMD) is a major risk factor for femoral head fracture, which in turn predisposes to functional impairment and femoral head ischaemia.^(8)^ Additionally, Looker *et al.* found a correlation between hip fracture incidence and femoral neck bone density.^(9)^ While early studies were equivocal about the effect of coffee intake on BMD, Tanski *et al.* found coffee intake to be a risk factor for BMD,^(10)^ Chua *et al.* found coffee intake to be a protective factor for BMD,^(11)^ while some other studies found no association between coffee intake and BMD. As a result, the relationship between coffee intake and FNBMD varies widely. The National Health and Nutrition Examination Survey (NHANES) is a cross-sectional survey conducted every 2 years since 1999 by the Centers for Disease Control and Prevention, to collect information on the health and nutritional status of the general population in the United States.^(12)^ The database contains a variety of data types, including demographic data, laboratory data, interview data, and restricted category data, as well as a stratified multi-stage sampling design that provides a better representation of the general US population.^(13,14)^ Mendelian randomisation (MR) is a statistical method that uses genetic variation as an instrumental variable, in which parental alleles are randomly assigned to offspring according to Mendelian inheritance laws, equivalent to random grouping in a randomised controlled trial.^(15)^ The instrumental variables used in MR studies are usually SNPs, which are reliably associated with exposure, do not vary with associated lifestyle or socio-economic factors, satisfy temporal sequential plausibility, and yield results with higher strength of evidence than observational studies and randomised controlled trials, on which they have been widely used in recent years to study causal associations in a variety of diseases.^(16,17)^ Thus, our study first explored the correlation between coffee intake and FNBMD using the NHANES database and further analysed the causality between the two using MR.

## Methods

### Study population in NHANES

In the observational study, we used data from NHANES 2005–2020 since these six cycles (2005–2006, 2007–2008, 2009–2010, 2013–2014, 2017–2018, and 2019–2020, respectively), specifically inquired information about FNBMD. FNBMD measurement using dual-energy X-ray (DXA) absorptiometry. Beginning in 2005, DXA scans have been administered in the NHANES mobile examination centre. Please see the NHANES database for details of the populations that can be measured (https://wwwn.cdc.gov/Nchs/Nhanes/2005-2006/DXXFEM_D.htm#DXXNKBMD).

### Coffee intake in NHANES

The collected 24-h dietary recall data in the survey cycle were used to estimate the caffeine intake in this study. For coffee intake data from codes beginning with ‘921’, we would use the average coffee consumption from two 24-h recalls. Combined with previous research, coffee intake was transformed into 6-ounce servings and categorised based on the number of cups consumed. At the same time, the cups/day are divided into five groups: no intake, <1, 1–<2, 2–<4, and 4+ cups/day.^(18)^

### Covariates used in NHANES

Based on previous studies, covariates that may confound the association between coffee intake and FNBMD were collected.^(19–21)^ The covariates of classified variables were sex (female, male), race/ethnicity (white, Mexican, black, other), education (under high school, high school or equivalent, above high school), marital status (married, living with partner, separated, divorced, widowed, never married), hypertension (no, yes), diabetes (no, yes), smoking (never, former, now), alcohol intake (never, former, heavy, mild, moderate), cancer (no, yes), and prednisone or cortisone (no, yes). The covariates of continuous variables were age (years), BMI (kg/m^2), total calcium (mg), poverty, physical activity (MET), and vitamin D (mg).

### Sources of MR

The MR study used two-sample MR. The exposure factor was coffee intake data from Ben Elsworth *et al.* sequenced in 2018, numbered ukb-b-5237, from the MRC-IEU Consortium, containing 428 860 sample sizes and 985 1867 SNPs. The outcome factor is the FNBMD data sequenced by Zheng *et al.* in 2015, number ieu-a-980, from the GEFOS Consortium, containing 32 735 sample sizes and 10 586 900 SNPs.^(22)^

### Statistical analyses

The NHANES data analysis refers to the NHANES statistical tutorial and follows its complex multi-stage probability sampling, weighting the sample. The weights were adjusted according to the method provided on the official NHANES website—the weighting variable was chosen as ‘wtdr2d’ and calculated as ‘1/6 * wtdr2d’. All analyses were performed under complex weighting. The continuous data were described using the mean and standard deviation (SD) and analysed using a *t*-test for statistical inference; the count data were described using the rate and compared with the chi-square test. Weight linear regression was used to explore the association between coffee intake with FNBMD. All covariates used the lowest quartile as the reference. Model 1 was a simple linear regression with coffee intake as the independent variable and FNBMD as the dependent variable. Model 2 was adjusted for age, sex, and race/ethnicity. Model 3 was further adjusted for all the covariates.

**Figure 1.**
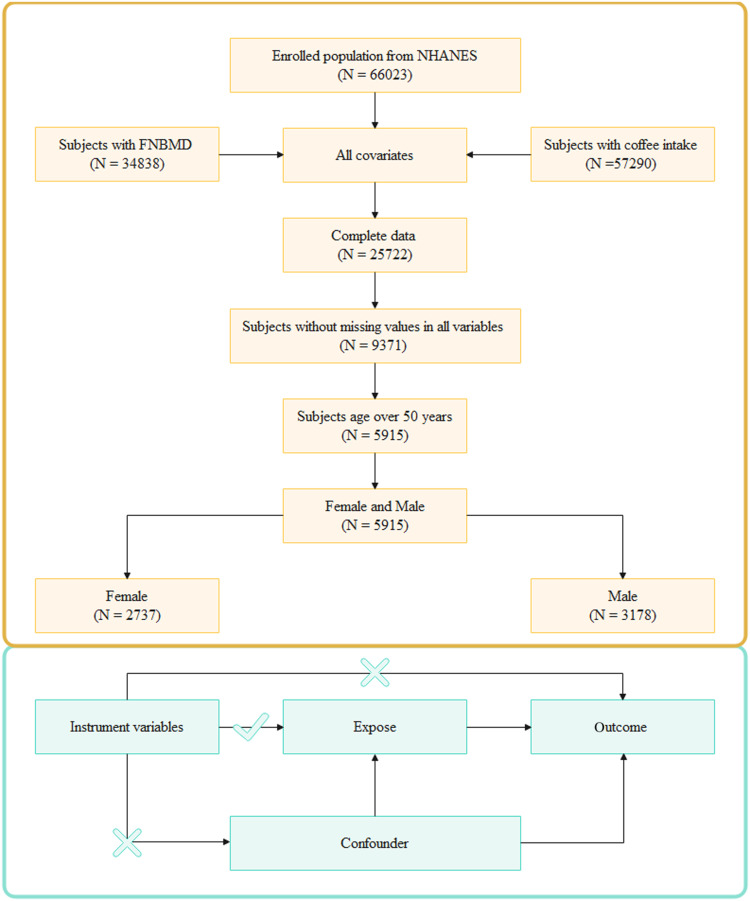
Study design overview. NHANES, National Health and Nutrition Examination Survey; FNBMD, femoral neck bone mineral density.

To explore the causal relationship between coffee intake and FNBMD, a two-sample MR approach was used. MR analysis was subject to three hypotheses: (1) instrumental variables for coffee intake were strongly associated with FNBMD; (2) instrumental variables for coffee intake were not associated with FNBMD; and (3) instrumental variables for coffee intake were not associated with confounders. SNPs with genome-wide significance that were independent of and strongly associated with coffee intake and FNBMD were selected as instrumental variables, an overview of the study design is shown in Fig. 1. The genome-wide significance parameter for coffee intake was set to P < 5×10^–8^, the linkage disequilibrium parameter (r2) was set to 0.001, and the genetic distance was set to 10 MB, to screen out instrumental variables with no linkage effect. The association between coffee intake and FNBMD was assessed using mainly an inverse variance weighting (IVW) method. However, the MR-Egger, WM, simple mode, and weighted mode methods were also used as supplementary methods. The method’s theory was described in previous studies. Heterogeneity was examined using the IVW and MR-Egger methods. Sensitivity analysis was performed using the leave-one-out method. Pleiotropy analysis was performed using the egger_intercept method. Finally, the strength of the association of the genetic instruments for each putative risk factor was quantified by the *F* statistic (*F* = β2/se2) for all SNPs, to assess the power of the SNPs.^(23)^

All statistical analyses were carried out using R (4.1.2) software, the ‘nhanesR’ package for NHANES data analysis, and the ‘TwoSampleMR’ package for MR analysis.

## Results

### Coffee intake and FNBMD in NHANES

There were 66 023 cases in the six cycles, of which 34 838 cases had FNBMD data, and a total of 57 290 cases had coffee data. After combining all covariates and excluding cases with missing values, there were 9 371 cases left. Finally, after excluding individuals younger than 50 years of age, there were a total of 5 915 cases, of which 3 178 were males and 2 737 were females. Weighted to represent 46 001 583 people, including 23 305 670 males and 22 695 913 females, the process is illustrated in Fig. 1.

The groups were formed by differences in intake of cups of coffee and analysed by gender in subgroups. In the overall population, there were differences between sex, BMI, race/ethnicity, marital status, poverty, smoking, alcohol intake, FNBMD, and physical activity; in the female population, there were differences between race/ethnicity, marital status, poverty, smoking, and alcohol intake, and in the male population, there were differences between race/ethnicity, marital status, poverty, smoking, cancer, and physical activity. Detailed results are shown in Tables 1–3.

**Table 1.** Weighted selected characteristics of study population in female and male, NHANES (weighted N = 46 001 583)

Characteristics	0 cup/day	<1 cup/day	1–<2 cup/day	2–<4 cup/day	4+ cup/day	*P*-value
Sex, %						< 0.001
Female	50.74	52.76	57.12	48.86	36.8	
Male	49.26	47.24	42.88	51.14	63.2	
Age, mean (SD)	62.02 (0.33)	61.68 (0.57)	62.64 (0.47)	62.10 (0.35)	61.09 (0.52)	0.24
BMI, mean (SD)	28.97 (0.15)	27.89 (0.51)	27.94 (0.33)	28.34 (0.31)	28.68 (0.26)	0.03
Race/ethnicity, %						< 0.001
White	73.95	60.36	72.52	84.53	90.78	
Mexican	3.89	8.84	6.08	3.9	2.38	
Black	10.82	11.12	9.68	4.03	1.87	
Other	11.33	19.69	11.72	7.54	4.96	
Education, %						0.14
Under high school	9.77	16.9	11.72	9.69	13.24	
High school or equivalent	25.94	25.11	23.26	22.04	26.18	
Above high school	64.29	57.98	65.02	68.28	60.57	
Marital status, %						< 0.001
Married	69.53	61.41	61.78	69.38	63.52	
Living with partner	1.93	2.76	4.34	2.16	2.11	
Separated	1.07	3.26	2.52	0.96	1.49	
Divorced	6.62	16.41	14.13	14.95	18.18	
Widowed	15	10.66	10.93	8.55	8.15	
Never married	5.85	5.49	6.29	4	6.55	
Poverty, mean (SD)	3.57 (0.06)	3.06 (0.14)	3.34 (0.12)	3.72 (0.08)	3.44 (0.14)	< 0.01
Smoking, %						< 0.001
Never	60.88	58.65	53.27	47.2	37.16	
Former	27.93	30.33	34.96	41.43	37.35	
Now	11.19	11.03	11.77	11.38	25.48	
Alcohol intake, %						0.005
Never	12.86	13.09	10.46	5.22	5.83	
Former	9.38	15.28	12.46	11.04	14.88	
Mild	46.86	46.5	51.39	52.53	44.78	
Moderate	19.12	12.97	15.46	19.98	19.67	
Heavy	11.79	12.16	10.23	11.22	14.84	
Diabetes, %						0.32
No	84.35	86.74	86.11	85.97	88.51	
Yes	15.65	13.26	13.89	14.03	11.49	
Hypertension, %						0.09
No	48.58	37.87	41.65	47.76	51.25	
Yes	51.42	62.13	58.35	52.24	48.75	
Cancer, %						0.26
No	82.42	84.72	84.93	79.14	82.78	
Yes	17.58	15.28	15.07	20.86	17.22	
FNBMD, mean (SD)	0.77 (0.00)	0.78 (0.01)	0.76 (0.01)	0.75 (0.01)	0.77 (0.01)	0.04
Physical activity, mean (SD)	4039.27 (134.66)	4709.26 (668.37)	3235.99 (278.78)	3450.84 (193.27)	3966.76 (242.93)	0.01
Total calcium, mean (SD)	939.99 (15.01)	933.99 (49.87)	917.30 (23.01)	924.66 (22.96)	998.02 (33.40)	0.42
Vitamin D, mean (SD)	4.89 (0.18)	5.91 (0.86)	4.67 (0.20)	4.60 (0.20)	4.81 (0.24)	0.44
Prednisone or cortisone, %						0.28
No	93.18	95.54	92.26	92.83	89.74	
Yes	6.82	4.46	7.74	7.17	10.26	

NHANES, National Health and Nutrition Examination Survey; SD, standard deviation; FNBMD, femoral neck bone mineral density.

**Table 2.** Weighted selected characteristics of study population in female, NHANES (weighted N = 22 695 913)

Characteristics	0 cup/day	<1 cup/day	1–<2 cup/day	2–<4 cup/day	4+ cup/day	*P*-value
Age, mean (SD)	62.34 (0.41)	62.52 (0.88)	63.09 (0.58)	62.32 (0.49)	62.28 (1.04)	0.82
BMI, mean (SD)	28.76 (0.24)	27.62 (0.62)	27.69 (0.48)	28.09 (0.43)	28.13 (0.59)	0.23
Race/ethnicity, %						< 0.0001
White	75.06	58.19	76.46	87.47	89.31	
Mexican	3.39	10.14	4.27	2.84	3.02	
Black	9.97	11.72	7.64	3.79	2.13	
Other	11.58	19.95	11.63	5.91	5.55	
Education, %						0.14
Under high school	9.43	16.97	10.27	8.38	14.58	
High school or equivalent	25.3	28.96	19.67	23.9	24.77	
Above high school	65.27	54.06	70.07	67.71	60.65	
Marital status, %						0.003
Married	64.19	53.06	59.39	63.83	48.72	
Living with partner	1.2	1.12	0.99	2.16	1.87	
Separated	1.1	3.18	2.19	0.84	2.22	
Divorced	8.03	18.26	16.49	17.25	20.28	
Widowed	20.26	17.39	14.93	13.59	17.88	
Never married	5.21	6.99	6.01	2.34	9.03	
Poverty, mean (SD)	3.54 (0.07)	3.01 (0.17)	3.37 (0.14)	3.71 (0.11)	3.16 (0.19)	0.002
Smoking, %						< 0.001
Never	65.45	65.93	62.45	51.63	44.55	
Former	25.09	26.29	28.74	36.9	25.37	
Now	9.45	7.78	8.81	11.47	30.08	
Alcohol intake, %						0.03
Never	18.43	20.73	14.93	7.54	9.74	
Former	9.18	15.29	12.49	8.55	14.34	
Mild	40.11	36.56	47.48	44.24	36.57	
Moderate	23.67	19.44	19.15	28.71	26.89	
Heavy	8.61	7.97	5.94	10.96	12.47	
Diabetes, %						0.76
No	13.83	15.6	14.32	11.81	10.87	
Yes	86.17	84.4	85.68	88.19	89.13	
Hypertension, %						0.11
No	49.78	38.61	45.47	47.8	59.12	
Yes	50.22	61.39	54.53	52.2	40.88	
Cancer, %						0.91
No	81.05	81.35	83.74	81.28	79.24	
Yes	18.95	18.65	16.26	18.72	20.76	
FNBMD, mean (SD)	0.72 (0.01)	0.73 (0.01)	0.72 (0.01)	0.70 (0.01)	0.72 (0.01)	0.28
Physical activity, mean (SD)	3192.65 (200.65)	3415.50 (663.71)	2503.82 (248.21)	3012.94 (258.91)	2898.99 (427.00)	0.27
Total calcium, mean (SD)	844.61 (17.87)	910.88 (101.46)	911.69 (31.81)	853.51 (38.11)	851.08 (39.39)	0.43
Vitamin D, mean (SD)	4.19 (0.16)	5.25 (0.71)	4.62 (0.25)	4.23 (0.30)	3.94 (0.33)	0.26
Prednisone or cortisone, %						0.2
No	92.19	94.49	91.9	91.14	85.25	
Yes	7.81	5.51	8.1	8.86	14.75	

NHANES, National Health and Nutrition Examination Survey; SD, standard deviation; FNBMD, femoral neck bone mineral density.

**Table 3.** Weighted selected characteristics of study population in male, NHANES (weighted N = 23 305 670)

Characteristics	0 cup/day	<1 cup/day	1–<2 cup/day	2–<4 cup/day	4+ cup/day	*P*-value
Age, mean (SD)	61.69 (0.38)	60.75 (0.70)	62.03 (0.73)	61.88 (0.50)	60.40 (0.61)	0.24
BMI, mean (SD)	29.18 (0.19)	28.18 (0.76)	28.27 (0.44)	28.58 (0.36)	29.00 (0.30)	0.18
Race/ethnicity, %						< 0.0001
White	72.8	62.78	67.29	81.72	91.64	
Mexican	4.42	7.38	8.49	4.91	2.01	
Black	11.7	10.44	12.38	4.27	1.72	
Other	11.08	19.41	11.84	9.11	4.63	
Education, %						0.36
Under high school	10.12	16.82	13.66	10.93	12.46	
High school or equivalent	26.6	20.81	28.05	20.26	27.01	
Above high school	63.27	62.36	58.29	68.81	60.53	
Marital status, %						< 0.001
Married	75.02	70.73	64.97	74.68	72.14	
Living with partner	2.69	4.59	8.81	2.16	2.25	
Separated	1.03	3.36	2.97	1.07	1.06	
Divorced	5.17	14.34	10.99	12.76	16.96	
Widowed	9.58	3.15	5.59	3.74	2.48	
Never married	6.5	3.82	6.67	5.59	5.11	
Poverty, mean (SD)	3.59 (0.07)	3.10 (0.23)	3.31 (0.16)	3.73 (0.09)	3.60 (0.15)	0.02
Smoking, %						< 0.001
Never	56.18	50.51	41.04	42.96	32.86	
Former	30.85	34.83	43.25	45.76	44.33	
Now	12.97	14.66	15.71	11.28	22.81	
Alcohol intake, %						0.15
Never	7.12	4.55	4.5	3.01	3.55	
Former	9.6	15.26	12.43	13.43	15.2	
Mild	53.81	57.6	56.59	60.45	49.57	
Moderate	14.42	5.75	10.53	11.64	15.46	
Heavy	15.05	16.84	15.95	11.48	16.21	
Diabetes, %						0.15
No	17.54	10.65	13.31	16.15	11.84	
Yes	82.46	89.35	86.69	83.85	88.16	
Hypertension, %						0.26
No	47.35	37.04	36.58	47.72	46.66	
Yes	52.65	62.96	63.42	52.28	53.34	
Cancer, %						0.04
No	83.83	88.49	86.51	77.09	84.83	
Yes	16.17	11.51	13.49	22.91	15.17	
FNBMD, mean (SD)	0.82 (0.01)	0.83 (0.02)	0.80 (0.01)	0.80 (0.01)	0.80 (0.01)	0.23
Physical activity, mean (SD)	4911.18 (259.55)	6154.10 (1154.04)	4211.27 (544.16)	3869.16 (264.44)	4588.52 (317.96)	0.01
Total calcium, mean (SD)	1038.22 (23.98)	959.80 (49.90)	924.76 (41.21)	992.63 (27.77)	1083.59 (47.53)	0.07
Vitamin D, mean (SD)	5.61 (0.28)	6.65 (1.65)	4.73 (0.30)	4.96 (0.24)	5.32 (0.29)	0.08
Prednisone or cortisone, %						0.72
No	94.21	96.71	92.74	94.44	92.36	
Yes	5.79	3.29	7.26	5.56	7.64	

NHANES, National Health and Nutrition Examination Survey; SD, standard deviation; FNBMD, femoral neck bone mineral density.

With no intake as a reference, univariate and multivariate linear regression showed that there was no significant difference in the overall population. Multifactorial linear regression showed that for ‘< 1’, ‘1–<2’, ‘2–<4’, and ‘4+’, the OR (95% CI) was 0.02 (–0.01, 0.04), 0.00 (–0.01, 0.02), –0.01 (–0.02, 0.00), and 0.00 (–0.01, 0.02), respectively. Both univariate and multivariate linear regression showed that there was no significant difference in the female population, and multifactorial linear regression showed that for ‘< 1’, ‘1–<2’, ‘2–<4’, and ‘4+’, the OR (95% CI) was 0.02 (–0.01, 0.05), 0.01 (0.00, 0.03), –0.01 (–0.03, 0.01), and 0.02 (0.00, 0.04), respectively. The male population showed no statistically significant differences in both univariate and multivariate linear regressions, and multifactorial linear regression showed that for ‘< 1’, ‘1–<2’, ‘2–<4’, and ‘4+’, the OR (95% CI) was 0.02 (–0.02, 0.05), –0.01 (–0.04, 0.01), 0.00 (–0.02, 0.01), and –0.01 (–0.03, 0.01), respectively. Detailed results are shown in Table 4.

**Table 4.** Linear regression of femoral neck bone mineral density risk across coffee consumption categories

Sex	Model	Coffee consumption (cup/day)				
		0	< 1	1–<2	2–<4	4+
Both	Model1	ref	0.01 (–0.02, 0.04)	–0.01 (–0.03, 0.00)	–0.02 (–0.03, 0.00)	0.00 (–0.01, 0.02)
	Model2	ref	0.01 (–0.02, 0.04)	–0.01 (–0.02, 0.01)	–0.01 (–0.02, 0.00)	0.00 (–0.02, 0.01)
	Model3	ref	0.02 (–0.01, 0.04)	0.00 (–0.01, 0.02)	–0.01 (–0.02, 0.00)	0.00 (–0.01, 0.02)
Male	Model1	ref	0.01 (–0.03, 0.05)	–0.02 (–0.04, 0.00)	–0.02 (–0.03, 0.00)	–0.02 (–0.04, 0.01)
	Model2	ref	0.01 (–0.03, 0.05)	–0.02 (–0.04, 0.00)	–0.01 (–0.03, 0.01)	–0.01 (–0.03, 0.01)
	Model3	ref	0.02 (–0.02, 0.05)	–0.01 (–0.04, 0.01)	0.00 (–0.02, 0.01)	–0.01 (–0.03, 0.01)
Female	Model1	ref	0.01 (–0.02, 0.04)	0.00 (–0.02, 0.02)	–0.02 (–0.04, 0.00)	0.00 (–0.02, 0.02)
	Model2	ref	0.01 (–0.03, 0.04)	0.01 (–0.01, 0.03)	–0.01 (–0.03, 0.00)	0.01 (–0.02, 0.03)
	Model3	ref	0.02 (–0.01, 0.05)	0.01 (0.00, 0.03)	–0.01 (–0.03, 0.01)	0.02 (0.00, 0.04)

Model 1 was a simple linear regression with coffee intake as the independent variable and femoral neck bone mineral density as the dependent variable. Model 2 was adjusted for age, sex, and race/ethnicity. Model 3 was further adjusted for all the covariates.

### Causal association between coffee intake and FNBMD in MR

**Fig. 2.**
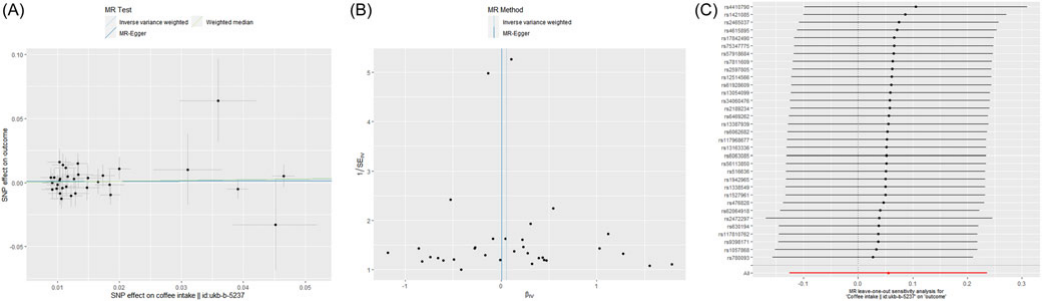
(A) Scatter plots, (B) funnel plot, and (C) forest map. MR, Mendelian randomisation.

Coffee intake was screened to include a total of thirty-three SNPs as instrumental variables. The results of MR showed consistent directions of analysis for the IVW, MR-Egger, WM, simple mode, and weighted mode methods, with the IVW results showing an OR (95% CI) of 1.06 (0.88–1.27), a *P*-value of 0.55, and an overall *F*-value of 80.31. The heterogeneity test showed a *P*-value of 0.62 for the IVW method and 0.57 for the MR-Egger method, revealing no heterogeneity. Sensitivity analysis showed no SNPs with a significant effect on the causal association estimates in the leave-one-out method. The results of the pleiotropy analysis showed a *P*-value of 0.76 for FNBMD, so there was no case of horizontal pleiotropy. Details are shown in Table 5 and Fig. 2.

**Table 5.** Mendelian randomisation estimates for the association between coffee intake and Femoral neck bone mineral density

Method	SNPs	B	P _Effect_	OR (95% CI)	P _Heterogeneity_	P _Intercept_
Inverse variance weighted	33	0.06	0.55	1.06 (0.88–1.27)	0.62	0.76
MR-Egger		0.01	0.97	1.01 (0.71–1.44)	0.57	
Weighted median		0.06	0.63	1.06 (0.83–1.37)		
Simple mode		0.20	0.44	1.22 (0.74–2.02)		
Weighted mode		0.03	0.84	1.07 (0.79–1.34)		

## Discussion

Our study first investigated the correlation between coffee intake and FNBMD using the NHANES database with six cycles (2005–2006, 2007–2008, 2009–2010, 2013–2014, 2017–2018, and 2019–2020) weighted by a total of 46 001 583 cases. In both the overall population and the female group, simple linear regression results showed a correlation between 2–<4 cups of coffee intake and FNBMD, but no correlation was found between coffee intake and FNBMD after adjusting for covariates (models 2 and 3). In the male group, both simple linear regression and multiple linear regression results showed no correlation between the two. Further applying a two-sample MR method with coffee intake data from the MRC-IEU Consortium and FNBMD data from the GEFOS Consortium, the IVW results showed an OR (95% CI) of 1.06 (0.88–1.27), and the results of the heterogeneity test, sensitivity analysis, and pleiotropy analysis showed no statistical differences, indicating that no causal relationship was found between coffee intake and FNBMD at the overall population level.

In previous studies, there was a large disagreement between coffee intake and BMD. In animal studies, Tsuang *et al.* found that caffeine had potentially harmful effects on osteoblast activity and could increase the rate of apoptosis in osteoblasts.^(24)^ Aparecida *et al.* found that coffee/caffeine intake had severe adverse effects on calcium metabolism in rats, resulting in elevated urinary and plasma calcium levels, which in turn reduced BMD.^(5)^ In a small sample of clinical studies, Feng *et al.* found that reducing coffee intake reduced the risk of osteoporosis^(25)^; Franca *et al.* found that over-caffeinated beverages may have a negative impact on BMD.^(26)^ In studies with large clinical samples, Choi *et al.* used Korean national data to find a trend towards increased age- and weight-adjusted BMD in the femoral neck, with increasing coffee intake,^(27)^ and a study from the Hong Kong Osteoporosis Study found a correlation between the presence of coffee-related serum metabolites and lower BMD in the femoral neck, over a 10-year period.^(28)^ However, in the present study, using data from a large clinical sample, there was no correlation between different levels of coffee intake and FNBMD after adjusting for covariates, either in the overall population or the female or male populations separately, in contrast to the findings of the aforementioned studies. Meanwhile, Lloyd *et al.* found no association between coffee intake and FNBMD or between coffee intake and longitudinal changes in FNBMD,^(9)^ and Wetmore *et al.* found no effect of coffee consumption on BMD in young women.^(29)^ The Framingham study, a long-term, prospective observational study, found that the association between coffee intake and hip fracture risk remained indirect^(30)^; Massey *et al.* found that the relationship between coffee intake and low BMD became less significant after adjusting for some of the covariates.^(31)^ Since coffee intake is usually confounded by more factors, a causal relationship could not be directly demonstrated, either with or without correlation. Therefore, our study combined a cross-sectional study with MR, which effectively circumvented the confounding of the results. The IVW results showed an OR (95% CI) of 1.06 (0.88–1.27) and a *P*-value of 0.55, demonstrating that there was no causal relationship between coffee intake and FNBMD. In contrast to the previous study in which Yuan *et al.* only used MR to explore the causal relationship between coffee intake and FNBMD,^(32)^ we first used cross-sectional large data for analysis, constructed models with different covariates at different gender levels, and used MR to further explore the causal relationship between the two. The results were validated in terms of correlation and causality, making them more robust.

Although the relationship between coffee intake and FNBMD is unclear, there are several possible scenarios. Coffee is a purine compound that affects osteoblast and osteoclast expression primarily through competitive inhibition of A_2A_ and A_2B_ receptors; its effect on BMD depends on its relative ability to block both receptors and the relative importance of the signalling pathways affected and may not directly affect changes in BMD.^(28)^ At the organismal level, coffee intake is mainly related to the efficiency of calcium absorption and excretion, resulting in increased urinary calcium excretion and increased blood calcium concentrations, but studies have shown that the increased urinary calcium excretion and increased blood calcium concentrations can also be counteracted by other substances consumed by the body, with no effect on BMD.^(2)^ At the coffee level, because there are more types of coffee, different coffee types and roasting levels lead to different chemical levels of coffee, which affect the final biopharmacological differences.^(18)^

However, our study still has some limitations: it was not possible to analyse different types of coffee to explore the effect of different types of coffee on the FNBMD, due to the limitations of the NHANES data source. Previous studies have found inconsistent effects of coffee intake on BMD in young, premenopausal, and menopausal women, but this study could not be analysed for that situation. Coffee intake in the NHANES database was collected on a patient-reported basis and may be affected by memory bias. Although the MR method was used in this study to investigate the causal relationship between the two, MR presupposes the direct existence of a linear relationship between the two, and if it does not exist, then MR is not applicable. BMD changes are a slow, cumulative process, and cross-sectional studies are subject to a variety of confounding factors that require more consideration.

## Conclusion

In summary, this study used cross-sectional studies and MR to demonstrate that there is no correlation or causal relationship between coffee intake and FNBMD, but prospective studies are still needed for confirmation.

## Data Availability

The data used in this study were publicly available.
